# Spatial and Socioeconomic Inequalities in Cesarean Section Deliveries in Urban Settings in Dakar, Senegal

**DOI:** 10.1007/s11524-024-00835-1

**Published:** 2024-03-20

**Authors:** Ibrahima Sy, Arsène Brunelle Sandie, Elhadji Malick Sylla, Birane Cissé, Ndèye Awa Fall, Mamadou Oumar Sow, Ndèye Bouri Silla, Cheikh Mbacké Faye, Aminata Niang Diène

**Affiliations:** 1 Department of Geography, University Cheikh Anta Diop of Dakar (UCAD), Dakar, Senegal; 2 African Population and Health Research Center (APHRC), West Africa Regional Office, Dakar, Senegal

**Keywords:** Cesarean section, Access, Inequalities, Dakar, Senegal

## Abstract

As part of an initiative aimed at reducing maternal and child mortality, Senegal implemented a policy of free Cesarean section (C-section) since 2005. Despite the implementation, C-section rates have remained low and significant large disparities in access, particularly in major cities such as Dakar. This paper aims to assess C-section rates and examines socioeconomic inequalities in C-section use in the Dakar region between 2005 and 2019. This study incorporates data from various sources, including the health routine data within District Health Information Software 2 (DHIS2) platform, government statistics on slum areas, and data from Demographic and Health Surveys (DHS). A geospatial analysis was conducted to identify locations of Comprehensive emergency obstetric and Newborn Care (CEmONC) services using the *Direction des Travaux Géographiques et Cartographiques* (DTGC) databases and satellite imagery from the Google Earth platform. The analytical approach encompassed univariate, bivariate, and multivariate analyses. The C-section rate fluctuated over the years, increasing from 11.1% in 2005 to 16.4% in 2011, declined to 9.8% in 2014, and then raised to 13.3% in 2019. The wealth tertile demonstrated a positive correlation with C-sections in urban areas of the Dakar region. Geospatial analyses revealed that women residing in slum areas were less likely to undergo C-section deliveries. These findings underscore the importance of public health policies extending beyond merely providing free C-section delivery services. Strategies that improve equitable access to C-section delivery services for women across all socioeconomic strata are needed, particularly targeting the poor women and those in urban slums.

**Supplementary Information:**

The online version contains supplementary material available at 10.1007/s11524-024-00835-1.

## Introduction

Limited access to Cesarean section (C-section) poses a substantial challenge to reducing maternal and neonatal mortalities, particularly among socially disadvantaged groups in sub-Saharan African (SSA). The SSA region accounts for two-thirds ((66%) of both maternal and under-5 deaths [1, 2]. The key indicators for maternal and child mortality highlight significant disparities between SSA and the rest of the world [3]. Indeed, with an estimated maternal mortality ratio of 629 deaths per 100,000 live births, the risk of death during pregnancy or childbirth remains very high in SSA (1 out of 16 pregnant women) compared to Europe (1 out of 2800 women).

C-section uptake delivery services are on the rise globally [4] playing a crucial role in the reduction of maternal and neonatal mortalities. In SSA, the increase in C-section uptake can be attributed to the adoption of free C-section policies implemented by several governments in early 2000s [5]. According to World Health Organization (WHO) recommendations, C-sections should be targeted toward women who are truly in need, with ideal rates between the range of 10 and 15% [6]. However, the C-section rate remains below 10% in West Africa, and high inequalities persist within countries where only 20% of the poorest women have access to C-section services [3–7].

Since the government’s adoption of the free C-section policy in all public health facilities in 2005 in Senegal, there has been noticeable increase in C-section delivery services uptake. This increase can be linked to the reduction of financial barriers to access to care [8]. The free C-section policy was implemented sequentially: (1) a total subsidy of C-sections in surgical health centers and hospitals in five pilot regions (Ziguinchor, Kolda, Tambacounda, Fatick, Matam); (2) a partial subsidy concerning only C-sections in other regions of Senegal outside Dakar; and (3) total subsidy in the Dakar region. The free policy was extended to the Dakar region from 2013 but clients were required to bear the cost of the surgery kit which cost about 40,000 CFA francs (about US$61) in addition to other expenses (consultation fee, prescriptions, etc.) estimated at 50,000 CFA francs (US$77) at public facilities [5–12]. These costs are sometimes beyond the reach of the poorest populations.

Despite these measures to facilitate access, the C-section uptake remained low nationally at 4%, substantially below the WHO’s recommended ideal range. That national rate also does not indicate the existence of large discrepancies in access to this obstetric service among regions (Dakar versus others) and within socioeconomic categories [9, 10].However, among private health facilities in the Dakar region, the C-section uptake was high (11%) and within the WHO recommended range, although there were significant variations by socioeconomic, geographic, and cultural factors [11]. This rate in Dakar city increased to 14.7% in 2018–2019 but social and spatial disparities were still evident.

In the Dakar region, studies on C-sections have focused heavily on the clinical aspect [10–13] and on evaluating its use in health facilities [14] and among women [15]. In contrast, inequalities in access to C-section that often affect poor women from disadvantaged backgrounds are not properly documented. Thus, this study aims to evaluate C-section rates in the Dakar region over time, to examine socio-spatial inequalities in access to C-sections, and to identify socioeconomic factors associated with the use of this surgical procedure in the urban areas of Dakar region.

## Materials and Methods

### Data Sources

Geospatial data encompassing regional, departmental, commune, and neighborhood boundaries in shapefile format were obtained from national specialized directorates such as the *Direction des Travaux Géographiques et Cartographiques* (DTGC), within the Ministry of Equipment and Transport; the National Agency for Spatial Planning; and the National Agency for Statistics and Demography. These cartographic resources were complemented by satellite imagery data, employed for the identification of slum areas within urban regions of the Dakar area through the Google Earth platform. The definition of slums in this study refers to areas or neighborhoods characterized by irregular, poorly developed, and precarious housing, occupied by poor populations. This type of poorly developed and informal housing represents about 30% of the inhabited areas in Dakar [16]. We also used data from the Ministry of Urban Planning, Housing and Living Environment, the national report “Cities without Slums of 2010,” a UN-Habitat report on the urban profile of Dakar of 2008 and the work of Borderon et al [17]. All these data were used to map the Dakar region and its slums.

C-section deliveries data for 2019 were obtained from the National Health Information System (NHIS) from the DHIS2 platform. These statistics are available at the district and public health facility levels.

Data used for the estimation of C-section rates over time and the determination of the associated factors were carried out within Standard (2005, 2011, and 2017) and continuous (2014, 2017, and 2019) Senegal Demographic and Health Survey (DHS) data. These data were obtained from Measure DHS program (https://dhsprogram.com/) after an approval request [36].

### Study Population

The study population comprised urban women of reproductive age residing in the identified slums in urban areas of the Dakar region. In order to examine C-section rates over time and associated factors, the study population was the subpopulation of women of childbearing age residing in urban areas of Dakar who delivered in a health facility. All the deliveries from the last 5 years before the survey was considered. Geospatial analyses were carried out to highlight the geographical location of health facilities delivering C-section according to the spatial distribution of slums housing poor pregnant women.

### Primary Outcome

The dependent variable in this study was the mode of delivery, defined as C-section delivery or normal vaginal delivery. The primary focus of the analysis of DHS data was C-section delivery among women living in urban areas of the Dakar region who delivered in a health facility. While in the geospatial analysis using DHIS2 data, the primary endpoint was the rate of C-section among women of reproductive age who live in the slums of urban areas in the Dakar region.

### Secondary Outcome

Emergency and elective C-section rates were also estimated to investigate the level of each type of C-section in Dakar. The type of C-section was captured based on the procedure’s timing; such data were collected only in the 2017 and 2019 DHS. The estimated rates of each type of C-section were determined by combining the two periods. An emergency C-section was defined as any procedure carried out after the delivery labor would have started, while elective C-section was defined as any procedure carried out before the onset of labor.

### Covariates

A set of covariates were considered for the multivariate analysis to determine factors associated with C-section uptake in the urban areas of the Dakar region using DHS data. The main covariate was the household wealth tertile index, determined from a composite indicator (including household assets such as television, radio, telephone, and air conditioner, and types of housing construction materials) based on multiple correspondence analysis. This continuous indicator was categorized into tertiles of three near-equal size groups ranked in ascending order: poor, medium, and rich. The other socioeconomic covariate considered was mother’s education level, categorized as none, primary, secondary, and higher. Sociodemographic covariates included mothers’ age group (15–24, 25–29, 30–39, and 40–49 years old) and marital status (married or single). Other control variables such as number of prenatal visits (none, 1–4, 8 and more), birth order (1, 2–3, 4 and more), type of health facility delivery (public or private), and finally the year of the survey (2005, 2011, 2014, 2017, and 2019).

### Data Analysis

#### Geospatial Analysis

Geospatial analyses involving geographic, sociodemographic, and health data were conducted using ArcGis and Qgis software. These analyses served several purposes including identification of slums and the health facilities offering C-sections and assessing geographical accessibility to Comprehensive emergency obstetric and Newborn Care (CEmONC) services according to the distance-reference tool of less than 2 km. Then, the C-section rate for 2019 was calculated in relation to all births recorded in health facilities providing this service, and the characteristics of the population living in the respective slums were identified by overlaying three layers of geographic information (slum location, population densities, and wealth level) with the goal of estimating the economic welfare of the populations. Using the Google Earth platform, we delimited the slums and their locations using criteria such as the type of buildings, building density, and names taken from the literature. Finally, indicators of C-section use were mapped at both the health facility and health district levels.

#### Statistical Analysis

C-section rates were calculated for each respective period, along with their corresponding 95% confidence intervals (95% CI) and then stratified according to the study covariates. The dataset for all time periods considered was then pooled for explanatory factor analysis using simple (to estimate unadjusted odds ratio (OR)) and multivariate (to estimate adjusted OR (aOR)) logistic regression. In the multivariate analyses, the main independent variables were wealth tertile and mother’s level of education. Then, multi-collinearity was checked among other variables to include only independent variables, while ensuring the main independent variables in the models. All analyses were weighted. Stata software version 15.1 [37], in particular the “svyest” module that takes into account the survey weights, was used for all statistical analyses.

## Results

### C-Section Access Rates According to Spatial Location and Socioeconomic Level

**Fig. 1:**
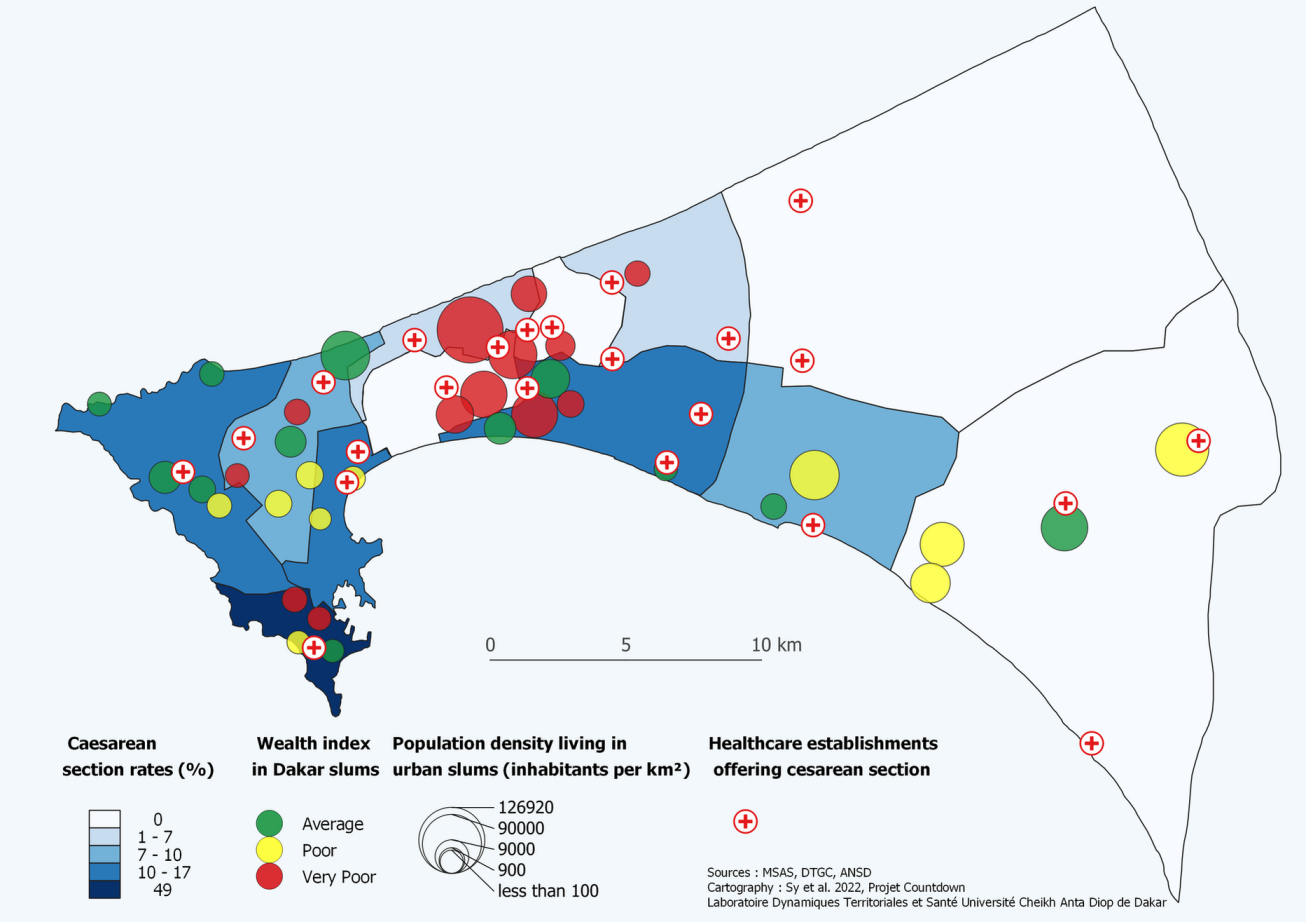
Cesarean section delivery rates in the slums of Dakar region by wealth tertile and spatial proximity to health facilities (DHIS2, 2019)

The geospatial analyses show a high number of health facilities providing C-sections services near the slums, which explains the good geographical accessibility within 2 km. In other words, nearly 75% of the slums with populations ranging from 100 to 126,900 inhabitants are located less than 2 km from a health facility providing C-sections (Fig. 1). However, this geographic accessibility does not necessarily determine the C-section rates of women residing in the slums which is lower.

**Fig. 2:**
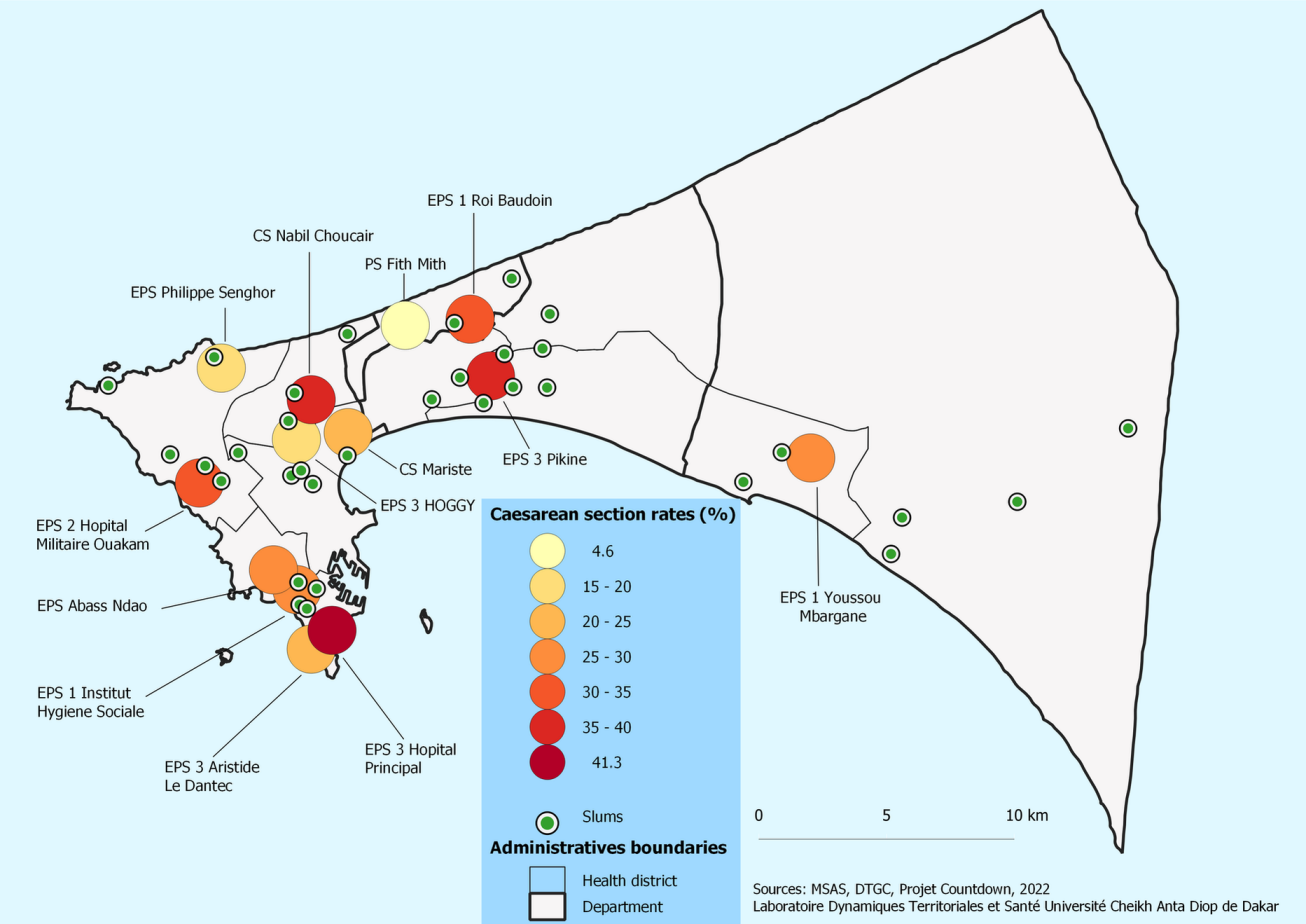
C-section delivery rate by health facility and location of slums in the Dakar region (DHIS2, 2019)

At the district level, the highest rates were found in Dakar South (49%), Dakar West (17%), Dakar East (14.4%), and Mbao (10%), while no C-section cases were recorded in Diamniadio and Sangalkam. As a result, the lowest C-section rates were recorded in areas with a high concentration of slums, which are home to a larger number of poor people (Fig. 2). However, at the health facility levels, nine public health facilities performing C-sections and located less than 2 km from slums performed this obstetrical service with rates ranging from 5% (113/2471) to 39% (1996/5070) of deliveries in 2019. Four health facilities located far from the slums performed C-sections with rates ranging from 17% (1335/7788) to 41% (743/1799) of deliveries.

### Trends and Distribution of C-Section Uptake According to Socioeconomic Characteristics (from DHS Data)

**Fig. 3:**
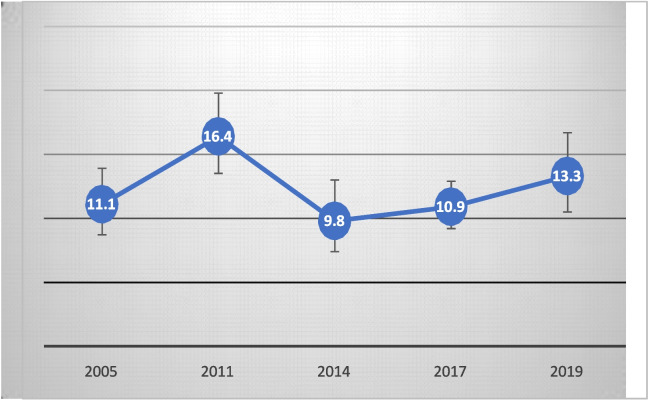
Trends in C-section delivery rates in the Dakar region from 2005 to 2019. Sources: Senegal Standard DHS 2005, 2011, and 2017; continuous DHS 2014 and 2019; sample sizes: *N* = 594, 619, 510, 1139, and 504, respectively, for 2005, 2011, 2014, 2017, and 2019

An analysis of the trend in C-section deliveries in urban areas of the Dakar region displayed in Fig. 3 shows a three-phase fluctuation in the rate: a gradual increase from 11.1 to 16.4% between 2005 and 2011; then, between 2011 and 2014, a significant decrease to 9.8%; and finally, a relative increase to 10.9% between 2014 and 2017 and 13.3% in 2019.

Table 1 presents C-section rates according to socioeconomic characteristics. Generally, for all respective periods, the rates were positively associated with the wealth tertile index, with higher prevalence among women from rich households. These differences were more noticeable and significant in 2011 with rates of 26.7% (95% CI = 20.8–33.7) among women from rich households to 10.3% (95% CI = 6.8–15.5) among women from poor households. In 2014 also, larger differences were observed, even though not significant, possibly due to low sample sizes. The C-section rate was 15.9% (95% CI = 10.8–22.8) among the richest versus 8.5% (95% CI = 4.9–14.1) and 4.8% (95% CI = 2.3–9.9) among the medium and poor, respectively. The woman’s level of education was also positively associated with C-section in urban areas of the Dakar region irrespective of the period of analysis. Prevalence was generally larger among women with higher educational levels. Similarly, the share of all C-section deliveries was higher in private health facilities than in public health facilities—particularly in 2014, where they comprised 29.0% (95% CI = 18.0–43.2) of all deliveries in private health facilities versus 7.1% (95% CI = 5.0–10.0) in public health facilities. Table 1 displayed the detailed variations of the C-section rate in the Dakar region over time according to socioeconomic and sociodemographic characteristics.

**Table 1:** Inequalities in the C-section rate in Dakar 2005–1019 according to socioeconomic and sociodemographic characteristics. Sources: Senegal Standard DHS 2005, 2011, and 2017; continuous DHS 2014 and 2019. Sample sizes: *N* = 594, 619, 510, 1139, and 504, respectively, for 2005, 2011, 2014, 2017, and 2019

	2005	2011	2014	2017	2019
	% (95% CI)	% (95% CI)	% (95% CI)	% (95% CI)	% (95% CI)
Overall rates	**11.1 (8.7–13.9)**	**16.4 (13.5–19.8)**	**9.8 (7.4–13.0)**	**10.9 (9.2–12.9)**	**13.3 (10.5–16.7)**
Wealth tertiles					
*Rich*	12.7 (8.7–18.2)	26.7 (20.8–33.7)	15.9 (10.8–22.8)	12.1 (9.2–15.8)	17.5 (12.3–24.3)
*Medium*	9.3 (5.8–14.6)	11.8 (7.9–17.3)	8.5 (4.9–14.1)	9.8 (7.1–13.4)	13.1 (8.7–19.3)
*Poor*	11.0 (7.3–16.3)	10.3 (6.8–15.5)	4.8 (2.3–9.9)	10.8 (7.9–14.5)	9.1 (5.5–14.7)
Mother’s level of education					
*No education*	8.5 (5.5–12.9)	13.8 (10.0–18.8)	4.0 (2.0–7.7)	10.5 (7.7–14.1)	9.4 (5.9–14.8)
*Primary*	12.0 (8.2–17.3)	15.5 (11.0–21.3)	5.8 (2.8–11.4)	7.4 (5.2–10.5)	9.7 (5.8–15.7)
*High school and above*	13.3 (8.7–19.9)	23.7 (16.6–32.5)	24.5 (17.3–33.4)	15.5 (12.0–19.7)	21.5 (15.6–28.8)
Type of health facility delivery					
*Public health facility*	10.4(8.0–13.3)	15.2 (12.3–18.7)	7.1 (5.0–10.0)	9.9 (8.1–11.9)	12.2 (9.4–15.7)
*Private health facility*	16.4(8.5–29.2)	25.6 (16.2–38.0)	29.0 (18.0–43.2)	19.0 (13.2–26.7)	23.1 (13.2–37.2)
Prenatal consultations					
*4* +	16.0(11.7–21.5)	19.2 (14.9–24.2)	10.1 (6.6–15.0)	14.0 (11.5–17.0)	15.6 (11.9–20.3)
*Less than 4*	8.0 (5.0–12.4)	17.2 (11.5–24.9)	8.6 (4.6–15.7)	4.8 (2.7–8.4)	12.3 (6.7–21.6)
Birth order					
*1st*	18.0 (12.6–25.0)	22.3 (16.5–29.4)	14.1 (9.0–21.5)	10.1 (7.3–13.9)	19.8 (14.2–26.9)
*2nd*–*3rd*	8.6 (5.5–13.1)	15.1 (10.8–20.8)	8.1 (4.9–12.9)	11.7 (9.0–15.1)	11.1 (7.4–16.3)
*4th and* +	7.9 (5.0–12.4)	12.4 (8.4–17.9)	8.0 (4.3–14.4)	10.6 (7.7–14.4)	8.6 (4.7–15.2)
Mother’s age					
*15*–*24*	10.3 (5.7–17.9)	15.5 (9.9–23.5)	8.6 (4.3–16.7)	7.9 (4.5–13.5)	9.8 (4.8–18.9)
*25*–*29*	12.8 (8.0–19.7)	13.2 (8.6–19.7)	7.5 (3.9–13.9)	4.4 (2.6–7.4)	11.7 (7.3–18.2)
*30*–*39*	10.1(7.1–14.1)	18.1 (13.7–23.6)	11.4 (7.5–16.9)	13.6 (10.9–16.9)	13.5 (9.4–18.9)
*40*–*49*	13.0(6.4–24.6)	21.0 (11.5–35.1)	12.8 (5.7–26.5)	18.9 (13.0–26.6)	22.9 (13.1–36.8)
Marital status					
*In union*	10.3 (7.9–13.2)	15.5 (12.6–18.9)	10.2 (7.5–13.6)	10.9 (9.1–13.0)	13.4 (10.5–17.1)
*Single*	19.0(10.4–32.0)	25.9 (15.3–40.4)	7.3 (2.2–21.5)	10.8 (6.0–18.7)	12.4 (5.8–24.6)

### Emergency and Elective C-Sections According to Socioeconomic Characteristics

Emergency and elective C-section rates were similar and estimated at 6.1% (95% CI = 4.8–7.7) and 6.2% (95% CI = 5.0–7.8) in 2017–2019, respectively. These rates were similar across the different socioeconomic categories considered as shown in Table 2.

**Table 2:** Emergency and elective C-section rates according to socioeconomic and sociodemographic characteristics. Source: continuous DHS 2014 and 2019. Sample size: *N* = 1165

	Emergency C-section % (95% CI)	Elective C-section % (95% CI)
Overall rates	6.1 (4.8–7.7)	6.2 (5.0–7.8)
Wealth tertiles		
*Rich*	8.1 (5.7–11.3)	7.8 (5.4–11.1)
*Medium*	6.0 (3.9–9.2)	6.1 (4.0–9.1)
*Poor*	4.1 (2.4–6.9)	4.8 (3.1–7.4)
Mother’s level of education		
*No education*	5.1 (3.2–8.0)	5.3 (3.5–7.9)
*Primary*	4.5 (2.7–7.3)	3.9 (2.3–6.5)
*Secondary* +	_8.8 (6.3_–_12.2)_	9.7 (7.0–13.3)
Type of health facility delivery		
*Public health facility*	5.2 (3.9–6.9)	5.6 (4.3–7.2)
*Private health facility*	12.7 (7.9–19.7)	11.1 (6.7–17.7)
Prenatal consultations		
*4* +	7.5 (5.7–9.8)	7.5 (5.7–9.7)
*Less than 4*	3.5 (1.6–7.5)	5.3 (2.8–9.7)
Birth order		
*1st*	8.5 (6.0–12.0)	6.8 (4.5–10.2)
*2nd*–*3rd*	5.9 (4.0–8.7)	5.8 (4.0–8.4)
*4th and* +	3.5 (1.9–6.3)	6.2 (4.0–9.4)
Mother’s age		
*15*–*24*	6.0 (3.2–11.1)	1.2 (0.3–5.0)
*25*–*29*	5.1 (3.0–8.3)	2.0 (0.9–4.3)
*30*–*39*	6.0 (4.3–8.5)	10.4 (7.9–13.4)
*40*–*49*	8.9 (4.9–15.8)	7.7 (4.2–13.7)
Marital status		
*Married/partnered*	6.4 (5.0–8.1)	6.3 (5.0–8.0)
*Single*	3.7 (1.3–9.7)	5.6 (2.6–11.7)

### Socioeconomic Factors Associated with the Use of C-Section Deliveries

The results of the multivariate analyses highlight the socioeconomic factors associated with C-section in Dakar (see Table 3). The year of the survey was associated with C-section use in the Dakar region. Overall, women were less likely to access C-sections in 2005, 2014, 2017, and 2019 than in 2011.

**Table 3:** Factors associated with C-section uptake in Dakar. Sources: combined Senegal Standard DHS 2005, 2011, and 2017 and continuous DHS 2014 and 2019, sample sizes: *N* = 3366

	Bivariate analysis		Multivariate analysis	
	Unadjusted OR	95% CI	Adjusted OR	95% CI
Survey year				
*2011*	Ref	Ref	Ref	Ref
*2005*	0.63*	0.45, 0.89	0.60**	0.42, 0.86
*2014*	0.56*	0.38, 0.82	0.53**	0.36, 0.78
*2017*	0.62*	0.46, 0.84	0.54***	0.40, 0.74
*2019*	0.78	0.55, 1.11	0.70	0.49, 1.00
Wealth tertile index				
*Rich*	Ref	Ref		
*Medium*	0.59***	0.46, 0.77	0.72*	0.55, 0.93
*Poor*	0.54***	0.41, 0.70	0.71*	0.54, 0.93
Mother’s level of education				
*No education*	Ref	Ref	Ref	Ref
*Primary*	1.03	0.78, 1.36	1.02	0.77, 1.35
*Secondary* +	2.21*	1.70, 2.87	2.09***	1.59, 2.74
Type of health facility delivery				
*Public health facility*	Ref	Ref		
*Private health facility*	2.35***	1.77, 3.12		
ANC visits				
*4* +	Ref	Ref		
*Less than 4*	0.59***	0.44, 0.78		
*Don’t know*	0.58***	0.44, 0.76		
*No antenatal visits*	1.59	0.30, 8.34		
Birth order				
*1st*	Ref	Ref		
*2nd*–*3rd*	0.66**	0.51, 0.85		
*4th and* +	0.58***	0.44, 0.76		
Mother’s age				
*15*–*24*	Ref	Ref	Ref	Ref
*25*–*29*	0.86	0.59, 1.24	0.84	0.58, 1.22
*30*–*39*	1.35	0.98, 1.85	1.38	1.00, 1.92
*40*–*49*	1.94**	1.30, 2.88	2.02***	1.35, 3.02
Marital status				
*In union*	Ref	Ref		
*Single*	1.20	0.84, 1.70		
Previous C-section				
*No*	Ref	Ref		
*Yes*	2.23***	1.181, 4.207		

*Ref*, reference category. (˟)*P* < 0.1, *P < 0.05, **P < 0.01, ***P < 0.001

The wealth tertile index was associated with C-section in the Dakar region. Children from poor and medium wealth households were less likely to be born by C-section compared to those from rich households (aOR = 0.71, 95% CI = (0.54–0.93) and aOR = 0.72, 95% CI = (0.55–0.93), respectively). Women with secondary and higher education levels were more likely to have a C-section compared to those with no education (aOR = 2.09; 95% CI = (1.59–2.74)). Women aged between 40 and 49 years were more likely to have a C-section than those aged 15–24 (aOR = 2.02; 95% CI = (1.35–3.02)). Women who gave birth in private health facilities were more likely to have a C-section than those who delivered in public facilities.

## Discussion

This study sought to analyze inequalities in the use of C-sections in the Dakar region based on a hypothesis anchored on geographic accessibility to health facilities providing these services and the sociodemographic and economic status of women’s households.

Although geospatial analyses show a high concentration of health facilities offering Comprehensive emergency obstetric and Newborn Care (CEmONC) near slums, geographic accessibility does not appear to have much influence on C-section rates for women residing in these types of urban settings [7, 18–21].

Despite the limited influence of the spatial proximity of the service, trends analysis shows that use of C-section has been broadly satisfactory in the Dakar region meeting WHO ideal range, including the first years of implementation of the free C-section policy in the Dakar region in 2013. The free C-section policy has certainly made the procedure more financially accessible to a greater number of women, thereby having a positive impact on the use of C-sections in the Dakar region. Consequently, the C-section rates recorded in the Dakar region (16.4% in 2011 and 13.3% in 2019) far exceed the national level and those of large urban areas in other countries in the West Africa sub-region [22, 23]. Moreover, a recent study shows a higher C-section rate in Dakar (26%) compared to the results of the DHS data analyzed and the threshold recommended by WHO [24].

One explanation of the increase in C-section deliveries in the Dakar region could be the increase in the level of income of urban households that is higher compared to other regions. The introduction of the free C-section policy in 2013 has had a positive impact on access for poor women [8–11].This result is consistent with that of Witter et al. [25], who showed that the exemption of costs related to C-section delivery had a positive impact in Senegal regardless of the socioeconomic status of the women households.

Our statistical analyses revealed significant association between the C-section uptake and factors such as women’s age, household wealth status, and the type of health facility. These results are consistent with other previous studies where C-section uptake was higher among women aged between 35 and 49 years, living in the rich households, and having at least secondary school education [24, 26–28]. Other studies have shown an increase in C-sections in younger mothers while others have shown a positive association between advanced maternal age and C-section. [24, 26, 29, 30]. In the Das et al. [30] study, for example, women aged 30 years and above had a higher probability (2.28) of having a C-section than younger women.

Our study results showed that C-section use is associated with household wealth with higher C-section uptake among women from rich households. A recent study at the national level shows that women in the two highest wealth quintiles are more likely to have C-section. [20]. Moreover, a study on inequalities in in-country C-section rates conducted in 72 low- and middle-income countries indicates that the use of C-section is lower among women from poor households and that, in addition, the increase in C-section rates does not imply a decrease in inequalities between the rich and the poor [31].

Our study highlighted a strong correlation between the type of health facility and the mode of delivery, showing that by giving birth in a private facility, a woman increases her probability of having a C-section by 34%. Another recent study correlated the overuse of C-sections with the private sector’s increasing share in medical and health care provision making this service a lucrative rather than a medically justified practice. In such a view, some health professionals therefore seek to change their patients’ treatment pathways to encourage the use of this practice in order to maximize their own income [32].

Other socioeconomic factors, including the respondents’ education, spouse’s education, place of residence, and wealth status, have an impact on C-section delivery among women in sub-Saharan Africa [27]. Moreover, use of this delivery option could be explained by low levels of births attended by skilled health personnel, especially in a poor or middle-income country [24, 27, 32].

Finally, sociocultural factors or aspects should also be highlighted such as physical appearance, the perception that vaginal delivery contributes to accelerating women’s aging, and the procedure saving time and therefore allowing women to quickly attend to other responsibilities in their lives, such as jobs and other children [8, 14, 33].

The study acknowledged certain limitations, primarily resulting from challenges in clearly differentiating the socioeconomic status of the household within slums. Further studies of socio-spatial inequalities in access to C-sections in the slums of the Dakar region could add to the empirical knowledge of access to comprehensive emergency maternal and newborn care among poor populations in African cities. The associations of C-sections with other sociocultural factors, including their acceptability and availability in public health facilities close to disadvantaged populations, particularly those living in the slums, remain unknown and constitute an interesting area to investigate for future studies.

## Conclusion

Over the period from 2005 to 2019, there was a general increase in both geographic accessibility to Comprehensive emergency obstetric and Newborn Care (CEmONC) services and the C-section rate in urban areas of the Dakar region. This upward trend in the rate from 2005 to 2011 shows that the free policy, effective in Dakar only from 2013, did not have a significant impact on the uptake of C-sections, since higher utilization rates were observed before the implementation of the policy. This suggests that recourse to C-sections during childbirth was a relatively common practice prior to the implementation of free policy in 2013, due to the deliberate choices of certain women with high socioeconomic income who have increasingly been opting for elective C-sections. Analysis of the statistical results shows that inequality factors in the use of C-sections were related to socioeconomic factors (wealth status and mother’s level of education) and sociodemographic characteristics (mother’s age). These results could suggest some bottlenecks in the implementation of the free C-section policy in Senegal in general and particularly in the urban areas of the Dakar region. Public health authorities should evaluate the impact of the free C-section policy on its access and monitor its effectiveness in the Dakar region for favorable C-section access and use. In summary, it is important for Senegal’s health authorities to ensure that rational and effective use of C-section deliveries in Dakar by targeting the categories of women who actually need this service and those who have more limited access.

## Supplementary Information

Below is the link to the electronic supplementary material.

Supplementary file1 (DOCX 21 KB)

## Data Availability

Data used in this study are available on a reasonable request to the corresponding author after countdown to 2030 approval.

## References

[1] World Health Organization. Trends in maternal mortality 2000 to 2017: estimates by WHO, UNICEF, UNFPA, World Bank Group and the United Nations Population Division. World Health Organization; 2019. Accessed October 27, 2023. https://iris.who.int/handle/10665/327595.

[2] UN-IGME-Child-Mortality-Report-2022.pdf. Accessed October 27, 2023. https://childmortality.org/wp-content/uploads/2023/01/UN-IGME-Child-Mortality-Report-2022.pdf.

[3] Zongo KA. Comment améliorer la qualité de la césarienne dans les pays d’Afrique sub-saharienne? Thèse de doctorat. Université Pierre et Marie Curie; Université Joseph Ki-Zerbo, Paris IV, Ouagadougou. 2015. p 93.

[4] Vogel JP, Betrán AP, Vindevoghel N, et al. Use of the Robson classification to assess caesarean section trends in 21 countries: a secondary analysis of two WHO multicountry surveys. *Lancet Glob Health*. 2015;3(5):260–70. 10.1016/S2214-109X(15)70094-X. 10.1016/S2214-109X(15)70094-X 25866355

[5] Witter S, Armar-Klemesu M, Dieng T. National fee exemption schemes for deliveries: comparing the recent experiences of Ghana and Senegal. In: Studies in HSO&P. 24:167–198.

[6] World Health Organization. WHO statement on Caesarean section rates. Accessed October 27, 2023. https://iris.who.int/bitstream/handle/10665/161442/WHO_RHR_15.02_eng.pdf?

[7] Dumont A, Guilmoto CZ. Trop et pas assez à la fois : le double fardeau de la césarienne. *Popul Soc*. 2020;581(9):1–4. 10.3917/popsoc.581.0001.

[8] Mbaye EM, Dumont A, Ridde V, Briand V. « En faire plus, pour gagner plus » : la pratique de la césarienne dans trois contextes d’exemption des paiements au Sénégal. *Santé Publique*. 2011;23(3):207. 10.3917/spub.113.0207. 21896215

[9] Ngom NF. L’assistance médicale à l’accouchement au Sénégal. Thèse de doctorat, Démographie. Université de Bordeaux. Français. 2016. p 373.

[10] Koulimaya-Gombet CE, Diouf AA, Diallo M, et al. Grossesse et accouchement des patientes ayant un antécédent de césarienne à Dakar : aspects épidémio-cliniques thérapeutiques et pronostiques. *Pan Afr Med J*. 2017;27(135):5. 10.11604/pamj.2017.27.135.11924. 10.11604/pamj.2017.27.135.11924 PMC5567968 28904664

[11] ANSD-UNICEF. Enquête par grappes à indicateurs multiples, situation des enfants et des femmes, Dakar Urbain 2015-2016, Sénégal. 2016. p 481.

[12] Witter S, Richard F, De Brouwere V. Les leçons apprises pour mieux intervenir dans le futur: comment réduire les barrières financières aux soins obstétricaux dans les pays à faibles ressources. In Richard F, Witter S, De Brouwere V editors. Réduire les barrières financières aux soins obstétricaux dans les pays à faibles ressources. 2008. pp 309–329. (Studies in Health Services Organisation & Policy; 25). ITGPress. https://lib.itg.be/pdf/itg/2008/2008shso0309.pdf.

[13] Faye Dieme M, Gassama O, Diouf AA, et al. Prise en charge et pronostic de l’accouchement en présentation du siège au centre de sante Nabil Choucair de Dakar (Sénégal). Journal de la SAGO (Gynécologie – Obstétrique et Santé de la Reproduction). 2018;19(2). Accessed June 9, 2022. http://jsago.org/index.php/jsago/article/view/43.

[14] Niang MM. Praevaluation de latique des césariennes dans un centre de santé de niveau 2 à Dakar. *J de la SAGO (Gynécol – Obstétr et Santé de la Reprod)*. 2017;18(1):12–6.

[15] Faye Dieme M, Moreira PM, Diouf AA, et al. Perception et vécu de la césarienne en milieu africain: enquête auprès de 280 patientes. *Annales de la SOGGO*. 2014;9(22):8.

[16] ONU-Habitat. Rapport du Sénégal sur la mise en œuvre du nouvel agenda urbain. 2021;[19066]. https://www.urbanagendaplatform.org/sites/default/files/2021-. Accessed 13 Aug 2022.

[17] Borderon M, Oliveau S, Machault V, Vignolles C, Lacaux JP, N’Donky A. Qualifier les espaces urbains à Dakar, Sénégal. *Cybergeo: Eur J Geogr*. Published online March 27, 2014.10.4000/cybergeo.26250

[18] ONU-Habitat. Profil du Secteur du Logement au Sénégal. 2012 pdf. Accessed October 27, 2023. https://unhabitat.org/sites/default/files/download-manager-.

[19] Banque Mondiale. Revue de l’urbanisation, villes émergentes pour un Sénégal émergent, RapportACS14161. https://documents1.worldbank.org/curated/en/900681468197983382/pdf/ACS14161-REVISED-FRENCH-WP-P124695-PUBLIC-Senegal-Urbanization-Review.pdf. Accessed 20 Jan 2023.

[20] Agence Nationale de la Statistique et de la Démographie (ANSD). Enquête Harmonisée Sur Les Conditions de Vie Des Ménages (EHCVM) Au Sénégal. Dakar, Sénégal ANSD, UEMOA, World Bank. 2021. p 181.

[21] Agence Nationale de la Statistique et de la Démographie (ANSD). Enquête Continue Sur La Prestation Des Services de Soins de Santé (ECPSS). Dakar, Sénégal ANSD, ICF. 2020. p 224.

[22] Institut National de la Statistique (INSTAT), Cellule de Planification et de Statistique Secteur Santé Développement Social et Promotion de la Famille (CPS/SS-DS-PF) et ICF. Enquête Démographique et de Santé 2018. USAID, INSTAT. 2018;24. Accessed October 27, 2023. https://dhsprogram.com/pubs/pdf/SR261/SR261.pdf.

[23] Institut National de la Statistique (INS) et ICF International. 2012. Enquête Démographique et de Santé et à Indicateurs Multiples de Côte d’Ivoire 2011-2012. Calverton, Maryland, USA : INS et ICF International. p 591.

[24] Stephie Gondjout T, Gassama O, Tété Diadhiou M, et al. Analysis of cesarean section indications according to the Robson classification in surgical maternities in Dakar, Senegal: about 9185 cases. *JGO*. 2020;8(5):135. 10.11648/j.jgo.20200805.12.

[25] Witter S, Armar-Klemesu M, Dieng T. Les systèmes nationaux d’exemption des coûts liés à l’accouchement: comparaison des expériences récentes du Ghana et du Sénégal, In : Studies in HSO&P, 25, 2008. pp 185–221.

[26] Dankwah E, Kirychuk S, Zeng W, Feng C, Farag M. Socioeconomic inequalities in the use of caesarean section delivery in Ghana: a cross-sectional study using nationally representative data. *Int J Equity Health*. 2019;18(1):162. 10.1186/s12939-019-1063-6. 31653255 10.1186/s12939-019-1063-6 PMC6814993

[27] Islam MA, Sathi NJ, Hossain MT, Jabbar A, Renzaho AMN, Islam SMS. Caesarean delivery and its association with educational attainment, wealth index, and place of residence in Sub-Saharan Africa: a meta-analysis. *Sci Rep*. 2022;12(1):5554. 10.1038/s41598-022-09567-1. 35365718 10.1038/s41598-022-09567-1 PMC8975863

[28] Diawara A, Sangho H, Tangara I, Cisse MO, Traoré MN, Konaté S. Complications Post césarienne et gratuité de la césarienne au Mali: cas d’un centre de santé de district. *Mali Méd*. 2014;XXIX(1):4. 30049141

[29] Philippe B. La prise en charge des femmes enceintes des communes isolées de Guyane, Mémoire de l’Ecole Nationale de Santé Publique, Rennes, France. 2001. p 93.

[30] Das P, Samad N, Sapkota A, et al. Prevalence and factors associated with Caesarean delivery in Nepal: evidence from a nationally representative sample. *Cureus*. 2021;13(12):15. 10.7759/cureus.20326. 10.7759/cureus.20326 PMC8743029 35028222

[31] Boatin AA, Schlotheuber A, Betran AP, et al. Within country inequalities in caesarean section rates: observational study of 72 low- and middle-income countries. In: BMJ 2018; 360:k55. 10.1136/bmj.k55. 10.1136/bmj.k55 PMC5782376 29367432

[32] Zehnati A, Bousmah MAQ, Abu-Zaineh M. Public-private differentials in health care delivery: the case of cesarean deliveries in Algeria. *Int J Health Econ Manag*. 2021;21(3):367–85. 10.1007/s10754-021-09300-x. 33786693 10.1007/s10754-021-09300-x

[33] Brugeilles C. L’accouchement par césarienne, un risque pour les droits reproductifs ? *Autrepart*. 2014;70(2):143–64. 10.3917/autr.070.0143.

