# Supplementary material for: Spatial and Socioeconomic Inequalities in Cesarean Section Deliveries in Urban Settings in Dakar, Senegal
Source: J Urban Health. 2024 Mar 20;101(Suppl 1):81–91. doi: 10.1007/s11524-024-00835-1 (PMC11602882; doi:10.1007/s11524-024-00835-1)
Supplement: Supplementary file 1 — Supplementary file1 (DOCX 21 KB) [file 11524_2024_835_MOESM1_ESM.docx]

**Appendices**

**Appendice 1 Distribution of sample characteristics according to sample year (Senegal Standard DHS 2005, 2011 and 2017 ; Continuous DHS 2014 and 2019)**

|  | **Survey Year** | | | | | |
| --- | --- | --- | --- | --- | --- | --- |
|  | **2005 % (n)** | **2011% (n)** | **2014% (n)** | **2017% (n)** | **2019% (n)** | **Overall% (n)** |
|  | **N=594** | **N=619** | **N=510** | **N=1139** | **N=504** | **N=3366** |
| **Mother's level of education** |  |  |  |  |  |  |
| *No edudation* | 36.2 (225) | 44.1 (266) | 43 (218) | 32.6 (369) | 36.5 (185) | 37.6 (1,263) |
| *Primary* | 40.6 (228) | 35.4 (224) | 31.3 (162) | 36.4 (411) | 31.8 (161) | 35.2 (1,186) |
| *Secondary +* | 23.3 (141) | 20.5 (129) | 25.7 (130) | 31 (359) | 31.7 (158) | 27.2 (917) |
| **Type of health facility delivery** |  |  |  |  |  |  |
| *Public health facility* | 88.7 (536) | 88.7 (549) | 87.5 (451) | 88.5 (1,005) | 89.4 (451) | 88.6 (2,992) |
| *Private health facility* | 11.3 (58) | 11.3 (70) | 12.5 (59) | 11.5 (134) | 10.6 (53) | 11.4 (374) |
| **ANC visits** |  |  |  |  |  |  |
| *Less than 4* | 32.8 (197) | 22.3 (138) | 27.9 (143) | 20.4 (233) | 19.5 (97) | 23.6 (808) |
| *4 +* | 37.8 (226) | 47.6 (296) | 43.8 (222) | 54.1 (619) | 58.9 (295) | 49.7 (1,658) |
| *don’t know* | 29.1 (169) | 29.8 (183) | 28.1 (144) | 25.4 (286) | 21.5 (111) | 26.5 (893) |
| *no antenatal visits* | 0.3 (2) | 0.3 (2) | 0.2 (1) | 0.1 (1) | 0.2 (1) | 0.2 (7) |
| **Birth Order** |  |  |  |  |  |  |
| *1* | 28.7 (172) | 31 (192) | 29.5 (150) | 29.3 (336) | 32.8 (162) | 30.1 (1,012) |
| *2-3* | 36.5 (214) | 34.4 (219) | 43.2 (219) | 40.7 (460) | 42.3 (211) | 39.6 (1,323) |
| *4 et +* | 34.9 (208) | 34.5 (208) | 27.3 (141) | 30 (343) | 24.9 (131) | 30.2 (1,031) |
| **Mother's age** |  |  |  |  |  |  |
| *15-24* | 20.8 (127) | 21.7 (136) | 20.2 (109) | 15.6 (172) | 17.2 (80) | 18.5 (624) |
| *25-29* | 25.2 (148) | 29.1 (176) | 29.1 (145) | 27.1 (310) | 28.4 (145) | 27.7 (924) |
| *30-39* | 45.2 (265) | 39.6 (251) | 40.8 (204) | 44.6 (512) | 43.9 (224) | 43 (1,456) |
| *40-49* | 8.9 (54) | 9.6 (56) | 9.8 (52) | 12.7 (145) | 10.5 (55) | 10.8 (362) |
| **Marital status** |  |  |  |  |  |  |
| *In union* | 91.3 (539) | 91.2 (564) | 88.3 (454) | 91 (1,033) | 88.5 (448) | 90.2 (3,038) |
| *Single* | 8.7 (55) | 8.8 (55) | 11.7 (56) | 9 (106) | 11.5 (56) | 9.8 (328) |
| **Previous CS** |  |  |  |  |  |  |
| *No* | 89 (522) | 84 (524) | 90.3 (463) | 88.7 (1,007) | 86.8 (439) | 87.8 (2,955) |
| *Yes* | 11 (72) | 16 (95) | 9.7 (47) | 11.3 (132) | 13.2 (65) | 12.2 (411) |

### Appendice 2: Chi 2 Independance test of independant variables two by two, for variable selection for the multivariate analysis (Data sources: Combined Senegal Standard DHS 2005, 2011, and 2017 and Continuous DHS 2014 and 2019; Sample sizes: N=3366)

|  | Survey year | Previous CS | ANC visits | Birth Order | Mother's level of education | Tertile Wealth Index | Mother's age | Marital status | Type of health facility delivery |
| --- | --- | --- | --- | --- | --- | --- | --- | --- | --- |
| Survey year |  | * | *** | * | *** | ns | ** | ns | ns |
| Previous CS | * |  | *** | *** | *** | *** | *** | ns | *** |
| ANC visits | *** | *** |  | *** | *** | *** | *** | *** | *** |
| Birth Order | * | *** | *** |  | *** | *** | *** | *** | *** |
| Mother's level of education | *** | *** | *** | *** |  | *** | ns | * | *** |
| Tertile Wealth Index | ns | *** | *** | *** | *** |  | ns | ns | *** |
| Mother's age | ** | *** | *** | *** | ns | ns |  | *** | ** |
| Marital status | ns | ns | *** | *** | * | ns | *** |  | ns |
| Type of health facility delivery | ns | *** | *** | *** | *** | *** | *** | ns |  |

(*˟)P < 0-1, *P < 0-05, **P < 0-01, ***P <0-001, ns : not significative*
